# Chronic Total Occlusions in Non-Infarct-Related Coronary Arteries and Long-Term Cardiovascular Mortality in Patients Receiving Percutaneous Coronary Intervention in Acute Coronary Syndromes

**DOI:** 10.3390/jcm13237094

**Published:** 2024-11-24

**Authors:** Irzal Hadžibegović, Ivana Jurin, Mihajlo Kovačić, Tomislav Letilović, Ante Lisičić, Aleksandar Blivajs, Domagoj Mišković, Anđela Jurišić, Igor Rudež, Šime Manola

**Affiliations:** 1 Department for Cardiovascular Diseases, Dubrava University Hospital, 10000 Zagreb, Croatia; ivanajurin1912@gmail.com (I.J.); antelisicic1980@gmail.com (A.L.); ablivajs@gmail.com (A.B.); andjelajurisic1@gmail.com (A.J.); simemanola@icloud.com (Š.M.); 2 Faculty of Dental Medicine and Health Care, Josip Juraj Strossmayer University, 31000 Osijek, Croatia; 3 Department for Internal Medicine, General Hospital Čakovec, 40000 Čakovec, Croatia; mihajlo1983@gmail.com; 4 Division of Cardiology, Department of Medicine, University Hospital Merkur, 10000 Zagreb, Croatia; tomislavletilovic@gmail.com; 5 School of Medicine, University of Zagreb, 10000 Zagreb, Croatia; 6 Department for Internal Medicine, General Hospital Slavonski Brod, 35000 Slavonski Brod, Croatia; domagoj1304@gmail.com; 7 Department of Cardiac and Transplantation Surgery, Dubrava University Hospital, 10000 Zagreb, Croatia; rudi@kbd.hr

**Keywords:** acute coronary syndrome, percutaneous coronary intervention, chronic total occlusion, complete revascularization, medical therapy adherence

## Abstract

**Background and aim**: Patients with non-infarct-related artery chronic total occlusion (non-IRA CTO) found during percutaneous coronary intervention (PCI) in acute coronary syndromes (ACSs) are not rare and have worse clinical outcomes. We aimed to analyze their long-term clinical outcomes in regard to clinical characteristics, revascularization strategies, and adherence to medical therapy. **Patients and methods**: The dual-center ACS registry of patients treated from Jan 2017 to May 2023 was used to identify 1950 patients with timely PCI in ACS who survived to discharge with documented adequate demographic, clinical, and angiographic characteristics, treatment strategies, and medical therapy adherence during a median follow-up time of 49 months. **Results**: There were 171 (9%) patients with non-IRA CTO. In comparison to patients without non-IRA CTO, they were older, with more diabetes mellitus (DM), higher Syntax scores (median 27.5 vs. 11.5), and lower left ventricular ejection fraction (LVEF) at discharge (median LVEF 50% vs. 55%). There was also a lower proportion of patients with high adherence to medical therapy (32% vs. 46%). Patients with non-IRA CTO had significantly higher cardiovascular mortality during follow-up (18% vs. 8%, RR 1.87, 95% CI 1.27–2.75). After adjusting for relevant clinical and treatment characteristics in a multivariate Cox regression analysis, only lower LVEF, worse renal function, the presence of DM, and lower adherence to medical therapy were independently associated with higher cardiovascular mortality during follow-up, with low adherence to medical therapy as the strongest predictor (RR 3.18, 95% CI 1.76–5.75). Time to cardiovascular death was significantly lower in patients who did not receive non-IRA CTO revascularization, although CTO revascularization did not show independent association with survival in the multivariate analysis. **Conclusions**: Patients with non-IRA CTO found during ACS treatment have more unfavorable clinical characteristics, worse adherence to medical therapy, and higher cardiovascular mortality. They need a more scrutinized approach during follow-up to increase adherence to optimal medical therapy and to receive revascularization of the non-IRA CTO whenever it is clinically indicated and reasonably achievable without excess risks.

## 1. Introduction

A coronary chronic total occlusion (CTO) is a progressive and mostly stable manifestation of coronary artery disease (CAD). It is defined as an occlusion of an epicardial coronary artery without antegrade blood flow being present for at least 3 months [1]. Previous studies have shown that 50–60% of patients with acute coronary syndromes (ACSs) have double-vessel or multivessel disease (MVD) and approximately 8–13% have a CTO in the non-infarct-related artery (non-IRA). In patients with ACS, the presence of a bystander true non-infarct-related artery chronic total occlusion (non-IRA CTO) is associated with increased mortality because of the reduced myocardial function and complication of fatal arrhythmias [2]. Indeed, one study has shown that CTO was associated with a poor prognosis only in patients with reduced baseline left ventricular ejection fraction (LVEF), but not in those with preserved LVEF [3]. The majority of data on the potential benefits of percutaneous coronary intervention (PCI) or revascularization of CTO in the past come from non-randomized, observational studies, which led to conflicting results.

The first, and so far, the only, randomized trial EXPLORE [4], included patients with acute myocardial infarction with ST elevation (STEMI) who were found to have a concurrent CTO in a non-IRA who were randomized to either CTO PCI or medical therapy alone showed no difference in LVEF, cardiac death, or major adverse cardiac event (MACE) between two groups. A recently published 10-year follow-up of the EXPLORE trial did not find an overall survival benefit after CTO PCI and no impact on MACEs [5]. However, it is unknown how adherent the patients were to the optimal medical therapy, which could also have influenced the results. Guideline-directed medical therapy (GDMT) in secondary prevention is an essential part of the strategy to achieve optimal clinical outcomes in patients with ACS and is highly recommended by evidence-based clinical guidelines [6].

One study has shown that GDMT at discharge as well as GDMT at 1 and 2 years was associated with lower 3-year risk of MACEs [7]. Although the observational studies [3,8] have shown the benefits of PCI CTO in non-IRAs, and the EXPLORE study [4] showed no difference in outcomes in STEMI patients, the optimal treatment strategy for these patients is still undefined by guidelines [6]. To the best of our knowledge, data about adherence to GDMT in these patients are scarce. Therefore, the aim of our study was to evaluate the prognostic impact of a non-IRA CTO after PCI in ACS according to the patients’ characteristics, treatment strategy selection (revascularization vs. medical therapy alone), and the adherence to GDMT.

## 2. Materials and Methods

Data were collected from an all-comer ACS registry at two Croatian centers, involving patients admitted between January 2017 and May 2023, who exhibited clear clinical, electrocardiographic, and laboratory indicators of ST elevation myocardial infarction (STEMI) or non-ST elevation acute coronary syndrome (NSTE-ACS), as defined by guidelines [7]. Patients were included in the study if they underwent emergent, urgent, or early coronary angiography and received PCI for the culprit lesion or lesions at the discretion of the operator during the same hospital stay and were discharged alive in stable condition.

In total, 1950 patients with appropriate inclusion criteria were found in the registry. We collected their demographic, clinical, laboratory, and angiographic data on the day of hospitalization and during coronary angiography and PCI and also collected data on treatment strategies and therapy adherence after discharge.

Multivessel disease (MVD) was defined as the presence of a stenosis of >50% found in at least one of the segments or important (>2.5 mm of diameter) branches of all three major epicardial coronary arteries. For patients with multivessel disease (MVD), the decision regarding ad hoc or elective total revascularization through PCI or coronary artery bypass graft (CABG) surgery was made by the operator or the institution’s Heart Team. Elective (postponed) total revascularization, whether by PCI or a CABG, was recorded only if performed within 12 months following ACS to avoid time-to-event bias. The complexity of coronary artery disease (CAD) was assessed using the Syntax score [9], calculated with the official Syntax score online calculator (https://syntaxscore.org/calculator/syntaxscore/frameset.htm (accessed on 1 June 2024).

CTO in a non-IRA was characterized as thrombolysis in myocardial infarction (TIMI) Grade 0 flow, with a suspected duration of coronary occlusion of at least 3 months, and by the typical appearance of a lesion, which includes visible mature collaterals on angiography and the absence of thrombus or staining at the proximal cap [10]. All patients received optimal medical therapy for acute coronary syndrome (ACS) as recommended by the guidelines [7] and underwent a comprehensive echocardiographic evaluation prior to discharge.

After discharge, patients were monitored through routine clinic visits and follow-up telephone calls. These calls were utilized to gather post-discharge information on clinical events, adherence to guideline-directed medical therapy (GDMT), and other significant details not captured during clinic visits. Adherence to GDMT was categorized as low, moderate, or high based on patient-reported actual doses and consistency of use of statins, dual antiplatelet therapy, or treatments for arterial hypertension, diabetes mellitus, and heart failure after discharge. If patients indicated both irregular use and dose reductions of any medication analyzed, their adherence was classified as low. Regular use with reduced dosing or inconsistent use of prescribed doses was considered moderate adherence, while consistent use of all prescribed doses of medications was classified as high adherence.

The primary clinical endpoint for this study was cardiovascular death, assessed through routine clinical visits and telephone calls. Cardiovascular death was defined as a death attributed to sudden death, myocardial infarction, stent thrombosis, heart failure, stroke, or pulmonary embolism. Non-cardiovascular deaths mainly included malignancies, bleeding, or trauma. Deaths with unknown causes or circumstances were categorized as other causes and did not qualify as the primary clinical endpoint.

This study was conducted as a part of the Cardiology Research Dubrava Prospective Registry (CaRDr) registered at ClinicalTrials.gov with ID NCT06090591. The research was carried out in line with the principles outlined in the Declaration of Helsinki and adhered to the ethical guidelines set by the committee overseeing patient data management. Approval was granted by the Ethics Committee of Dubrava University Hospital (protocol code 2020/2409-08, approved on 24 September 2020).

## 3. Statistical Analysis

The Kolmogorov–Smirnov test was used to assess the normality of distribution. All continuous numerical variables demonstrated a non-normal distribution and were presented as median and interquartile range. The Mann–Whitney test evaluated significant differences between two groups. Categorical variables were reported as frequencies and percentages, with the χ^2^ test applied to examine significant differences in proportions between the two groups. Event-free survival differences were analyzed using the Kaplan–Meier method, and survival curves were compared univariately with the Mantel–Cox log-rank test. Multivariate regression analyses were conducted using Cox regression. All variables that showed significant differences (a total of 14 from Table 1 and Table 2) regarding non-IRA CTO identified on coronary angiography were included in the main multivariate regression equation. The final Cox regression model for predicting cardiovascular mortality related to the presence of non-IRA CTO was selected through a stepwise approach, utilizing “entry” and “stay” criteria of *p* ≤ 0.10. The same criteria were applied when calculating the regression model for cardiovascular mortality prediction within the subgroup of patients with non-IRA CTO in relation to subsequent CTO revascularization. The statistical significance level was set at *p* < 0.05, and Bonferroni correction for multiple simultaneous comparisons was applied as needed. The analysis was conducted using IBM SPSS software, version 19 (IBM, Armonk, NY, USA).

## 4. Results

### 4.1. Overall Characteristics, Risk Factors, and Treatment

Among a total of 1950 patients included, there were 1177 (60%) patients with ST elevation myocardial infarction (STEMI) and 773 (40%) patients with non-ST elevation acute coronary syndrome (NSTE-ACS). The patients were mostly male (71%), with a median age of 63 years (IQR 17 years). The median Syntax score was 12 (IQR 12.5), with 365 (19%) patients having MVD. Total revascularization by PCI or a CABG within 12 months of PCI in a qualifying ACS event was achieved in 1549 (79%) patients.

There were 171 (9%) patients with non-IRA CTO determined on initial coronary angiography performed during the invasive treatment of ACS. Among them, 57/171 (33%) patients had a CTO of the left anterior descendent artery (LAD). Patients with non-IRA CTO differed significantly in many relevant demographic and clinical characteristics from patients without CTO in a non-IRA. They were predominantly male, significantly older, with significantly lower creatine clearance levels (CrCl), and with significantly more cases of arterial hypertension (AHT), diabetes mellitus (DM), peripheral artery disease (PAD), previous MI, PCI, and CABG, and psychological disorders requiring medical attention. Patients with non-IRA CTO presented significantly more frequently with NSTE-ACS. Also, CAD complexity measured by Syntax score was significantly higher among patients with non-IRA CTO (*p* < 0.05 for all comparisons; Table 1), with significantly more patients having multivessel disease. There were no significant differences in other clinical characteristics or in the proportion of patients with an LAD as an IRA, cardiogenic shock and/or resuscitation at presentation, and vascular access (Table 1).

The proportion of patients who received total revascularization within 12 months of initial ACS and culprit lesion management was significantly lower in the non-IRA CTO group. Among the 171 patients with non-IRA CTO, in 51 (30%) patients, CTO was revascularized within 12 months, mainly by PCI (45/51 patients). In the case of multivessel disease as defined in this study, total revascularization was achieved by PCI in only 31/95 (32%) patients with non-IRA CTO and in 220/279 (79%) patients without non-IRA CTO. Also, there were 6/95 (6%) patients among the non-IRA CTO group that achieved complete revascularization with a CABG, in comparison to 24/279 (9%) patients with a CABG as a strategy of complete revascularization among patients with multivessel disease without CTO. Although there were 57/171 (33%) patients with left anterior descendent non-IRA CTO, 25/51 (49%) of the revascularized non-IRA CTOs were in the left anterior descendent artery. Patients with non-IRA CTO had significantly lower LVEF at discharge. All patients received dual antiplatelet therapy (DAPT) and statin therapy at discharge, except a minority of patients with contraindications or at physician discretion at discharge. There were significantly more patients receiving clopidogrel as a part of DAPT in the non-IRA CTO group. Also, there were significantly more patients with low or moderate adherence to medical therapy in the non-IRA CTO group (Table 2).

### 4.2. Clinical Outcome Associated with Non-IRA CTO Found on Coronary Angiography

The median follow-up for the whole cohort was 49 months. During the follow-up period, 171 patients (9%) from the entire cohort died from cardiovascular causes. The rate of cardiovascular mortality during long-term follow-up was higher in the non-IRA CTO group (18% compared to 8%), with an unadjusted relative risk of 1.87 for cardiovascular death (95% CI 1.27–2.75, *p* = 0.002). After adjusting for non-IRA CTO and all demographic and clinical factors from Table 1 and Table 2 that significantly impacted cardiovascular mortality in the master regression equation (specifically age, creatinine clearance (CrCl), diabetes mellitus, peripheral artery disease, Syntax score, ejection fraction at discharge (EFLV), and adherence to medical therapy), only lower EFLV at discharge, lower CrCl, diabetes mellitus, and low adherence to medical therapy remained independently associated with cardiovascular death during follow-up. The presence of non-IRA CTO and high Syntax scores did not show a significant independent association with cardiovascular mortality over this period. Low adherence to medical therapy had the strongest independent association with cardiovascular mortality, although it was accompanied by a relatively wide 95% confidence interval (RR 3.18, 95% CI 1.76–5.75; Table 3).

### 4.3. Non-IRA CTO Revascularization and Cardiovascular Mortality

**Figure 1:**
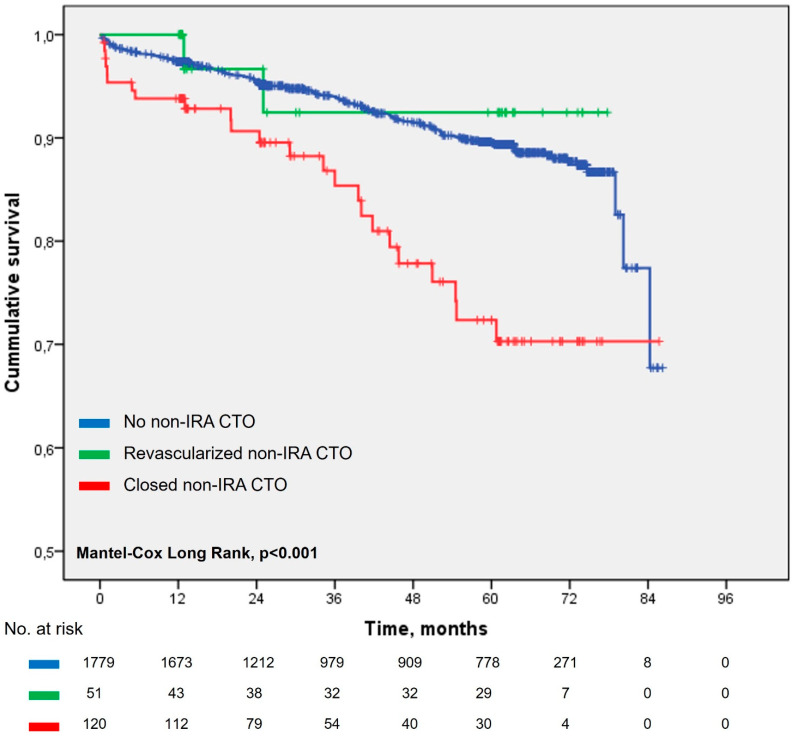
Long-term survival after percutaneous coronary intervention in acute coronary syndrome regarding the status of chronic total occlusion found on initial coronary angiography.

For the univariate time to cardiovascular death analysis, patients were divided into three groups: 1779 patients without non-IRA CTO, 51 patients with non-IRA CTO that was subsequently revascularized, and 120 patients with non-IRA CTO that was left closed (not revascularized). Patients with non-IRA CTO that was left closed during follow-up had significantly shorter time to cardiovascular death, in comparison to patients without non-IRA CTO and patients that received non-IRA CTO revascularization (Mantel–Cox log rank, *p* < 0.001; Figure 1).

A sub-analysis of 171 patients with non-IRA CTO regarding CTO revascularization did not show any significant differences in important demographic and clinical variables, except for cardiovascular death during follow-up. Patients who received subsequent non-IRA CTO revascularization were younger, with more cases of diabetes mellitus, and with higher CrCl, but the differences did not reach statistical significance within the non-IRA CTO subgroup (Table 4). They also had higher LVEF at discharge and more patients with high adherence to medical therapy (47% vs. 27%, respectively), but the differences did not reach statistical significance. However, there was a significantly lower proportion of patients with cardiovascular death during follow-up in comparison to patients who did not receive revascularization of the non-IRA CTO (Table 5). Their unadjusted relative risk for cardiovascular mortality was 0.32, 95% CI 0.10—1.02 (*p* = 0.055); a reduction in relative risk for cardiovascular death of 68% was found after successful CTO revascularization, almost reaching statistical significance. After adjusting for variables that fit the “entry” and “stay” criteria among this subgroup, only higher age and lower EFLV at discharge showed significant independent association with cardiovascular mortality during follow-up among the non-IRA CTO subgroup only, whereas non-IRA CTO revascularization almost reached statistical significance, but with a very wide confidence interval (Table 6).

## 5. Discussion

Our study confirmed that patients with CTO in a non-IRA found during PCI in ACS were older and had more unfavorable clinical factors associated with poorer outcomes, such as DM, worse renal function, and lower LVEF. Also, they had significantly lower adherence to GDMT, which is a novel finding, and it was the strongest independent factor related to cardiovascular death. In the case of non-IRA CTO, complete revascularization was achieved significantly less frequently, and it was achieved mainly by PCI, most likely because of more unfavorable demographic and clinical characteristics. Subsequent revascularization of non-IRA CTO was associated with significantly longer time to survival in regard to cardiovascular death (Figure 1). However, multivariate analysis in the non-IRA CTO subgroup showed that only younger age and better LVEF exhibited significant independent association with a better outcome. Although CTO revascularization showed a marked reduction in the adjusted relative risk of cardiovascular mortality (Table 6), it was accompanied by evidently wide 95% confidence intervals and did not reach statistical significance for independent impact on cardiovascular mortality. A similar result of the multivariate analysis was also observed for non-IRA CTO revascularization of the left anterior descendent artery. That could be explained by a relatively small absolute number of patients achieving revascularization of the non-IRA CTO within 12 months of PCI in ACS, as it was seen in other similar studies [5].

In our cohort, the timing of CTO revascularization following primary PCI for acute coronary syndrome (ACS) was determined at the discretion of the operator or the institution’s Heart Team and was recorded only if performed within 12 months of ACS. If patients underwent subsequent revascularization after 12 months post-admission, it was not categorized as total revascularization to avoid time-to-event bias. The optimal timing for staged CTO-PCI after primary PCI for ACS remains unclear. Some patients in this cohort did receive revascularization after 12 months, but they were classified as lacking non-IRA CTO revascularization. It could be argued that if more patients had successful CTO revascularization in our cohort, the results of the multivariate analyses might differ somewhat from those presented, given the relatively strong indication of benefits associated with both successful CTO revascularization and optimal adherence to guideline-directed medical therapy (GDMT).

A notable finding in our study is that patients who underwent CTO revascularization demonstrated higher adherence to guideline-directed medical therapy (GDMT), although this difference did not achieve statistical significance (Table 5). Medication non-adherence after PCI and myocardial infarction continues to be a significant issue, with suboptimal adherence observed across several key medication classes, including statins and antiplatelet agents [11]. Our all-comer ACS registry is a prospective, observational registry, and patients were regularly contacted regarding adherence to therapy. Since revascularization of CTO of a non-IRA is an elective procedure, special emphasis was placed on shared decision making on whether to perform the procedure or not. We believe that shared decision making between patients and healthcare professionals is considered a foremost process in defining the appropriate therapeutic pathway. Invasive treatment of residual CAD with either a CABG or PCI after the stabilization of ACS carries certain risks, but also improves angina-related health status [12]. The CLARIFY registry found that many patients with chronic coronary syndrome (CCS) and angina experience a resolution of symptoms over time, often without changes in treatment or a revascularization strategy, and experience good outcomes [13]. Considering that the patients who were revascularized in our cohort were more adherent to GDMT, it could be argued that they were probably more symptomatic and more susceptible to treatment strategies proposed by medical professionals during follow-up. In parallel, it could also be argued that the patients who were not revascularized for the CTO of a non-IRA were less symptomatic, older, with lower LVEF, and less active, hence less prone to adhere to proposed procedures or even GMDT. That could potentially lead to worse outcomes, as was shown in our study. It is known that effective and clear physician–patient communication is imperative to achieve and maintain the best therapeutic benefits, which is in line with our results [14]. After publication of the ISCHEMIA trial results [15], initial conservative medical management of CCS patients started to be generally preferred. However, several meta-analyses have confirmed consistently greater freedom from ACS and anginal symptoms after revascularization compared with GDMT alone [16,17,18]. Based on our study results, we believe the optimal treatment strategy is revascularization of non-IRA CTO if reasonably achievable and clinically indicated, in combination with high adherence to GDMT. Also, the final revascularization strategy should be based on coronary anatomy and on the decision of an institutional Heart Team.

There are several limitations to this study. First, this is a moderate-sized, dual-center, observational, all-comer registry with a relatively small number of patients with non-IRA CTO found and subsequently revascularized. The selection of patients for CTO-PCI or CABG was observed and left to preferences of patients and the discretion of physicians after discharge, so we cannot exclude the possibility of selection bias. Secondly, the myocardial viability tests were not routinely performed in all patients, and they were not documented in the patients’ characteristics. That might have influenced the clinical outcomes because the selection of patients for subsequent revascularization was influenced by many factors, including symptoms, echocardiography findings, ECG stress tests, and also viability tests that were not widely available and documented. Recent studies found an important role of cardiac magnetic resonance (CMR) in assessing viability and functional left ventricular disability potentially reversed with subsequent total revascularization [19]. In regard to therapy, we did not document the severity of symptoms and reasons for changes in adherence to medical therapy, which could also influence treatment strategies. Also, there are relatively novel therapies that could affect patients’ myocardial recovery after PCI in ACS, especially in the presence of a bystander CTO [20]. However, we did not include that in our sub-analyses because of the relatively late introduction of those therapies in our routine clinical practice due to reimbursement issues. CTO PCI often requires additional imaging, either by intravascular ultrasound or optical coherence tomography, to increase the interventional success rate and to obtain optimal results in the long-term follow-up [21]. We did not collect data on the use of additional imaging during total revascularization by PCI. Future studies with imaging-guided total revascularization in patients with ACS and MVD with concomitant CTO are needed to clarify the advantages of additional imaging in that setting.

In conclusion, we found that every tenth patient with PCI in ACS has non-IRA CTO confirmed at baseline coronary angiography. Because of more unfavorable clinical characteristics and worse adherence to medical therapy, those patients need to be followed with more scrutiny and offered more chances to increase adherence to GMDT and to receive revascularization of the non-IRA CTO despite the reported severity of symptoms if it is clinically indicated and reasonably achievable without excess risks. Education on the importance of GMDT should be performed during a hospital stay and at each visit, and the number of visits for those patients during the first 12 months should be increased. We believe that small absolute numbers and the heterogeneity of patients receiving appropriate revascularization of CTO lesions after successful treatment of ACS led to most studies not proving significantly better long-term results after CTO revascularization, and that the strategy of emphasizing high adherence to GDMT together with appropriate revascularization according to anatomical and clinical features probably offer the best long-term results in both acute and chronic coronary syndromes.

## Figures and Tables

**Table 1:** Clinical characteristics of patients with ACS in regard to non-IRA CTO found on initial coronary angiography and PCI.

Clinical Characteristic, Median (IQR) or Number (%)		Non-IRA CTO on Coronary Angiography		*p* Value (Mann–Whitney or χ^2^ Test)
		Yes *n*= 171	No *n* = 1779	
Age, years		68 (17)	63 (16)	<0.001
Male sex		137 (80)	1247 (70)	0.003
Creatinine clearance, ml/min		71 (38)	82 (31)	<0.001
Arterial hypertension		140 (81)	1299 (73)	0.006
Diabetes mellitus		60 (35)	389 (22)	<0.001
LDL-C, mmol/L		3.3 (1.8)	3.5 (1.6)	0.062
Peripheral artery disease		40 (23)	187 (11)	<0.001
Previous myocardial infarction		45 (26)	205 (12)	<0.001
Previous PCI		37 (22)	198 (11)	<0.001
Previous CABG		12 (7)	27 (2)	<0.001
Previous stroke		13 (8)	90 (5)	0.110
Chronic obstructive pulmonary disease		10 (6)	29 (2)	0.592
Current smoking		83 (48)	867 (49)	0.863
Atrial fibrillation, any form		18 (11)	124 (7)	0.250
Body mass index, kg/m^2^		29.3 (5.3)	28.4 (5.6)	0.243
ACS type	STEMI	86 (50)	1091 (61)	0.021
	NSTE-ACS	85 (50)	687 (39)	
Wrist vascular access		138 (81)	1494 (84)	0.213
Left anterior descendent as IRA		71 (42)	711 (40)	0.082
Syntax score		27.5 (14.5)	11.5 (11)	<0.001
Multivessel disease		95 (56)	279 (16)	<0.001
Cardiogenic shock and/or cardiopulmonary resuscitation		15 (9)	104 (6)	0.548

Non-IRA CTO—non-infarction-related artery chronic total occlusion, PCI—percutaneous coronary intervention, IQR—interquartile range, CABG—coronary artery bypass graft, LDL-C—low-density lipoprotein cholesterol, STEMI—ST elevation myocardial infarction, NSTE-ACS—non-ST elevation acute coronary syndrome, IRA—infarct-related artery.

**Table 2:** Treatment strategies and long-term outcomes after discharge regarding non-IRA CTO found on initial coronary angiography for ACS.

Treatment and Outcome Variables, Number (%) or Median (IQR)		Non-IRA CTO on Coronary Angiography		*p* Value (χ^2^ or Mann–Whitney Test)
		Yes *n* = 171	No *n* = 1779	
Complete revascularization		51 (30)	1498 (84)	<0.001
LVEF at discharge, %		50 (13)	55 (16)	<0.001
DAPT at discharge	Ticagrelor	110 (64)	1262 (71)	0.025
	Prasugrel	18 (10)	238 (13)	
	Clopidogrel	42 (25)	274 (16)	
	No DAPT	1 (1)	5 (0)	
Statin at discharge	Maximal dose	161 (94)	1689 (95)	0.853
	Submaximal dose	9 (5)	71 (4)	
	No statin	1 (1)	19 (1)	
Adherence to medical therapy after discharge	Low	44 (26)	356 (20)	0.004
	Moderate	72 (42)	605 (34)	
	High	54 (32)	818 (46)	
Cardiovascular death during follow-up		26 (18)	145 (8)	0.002

IQR—interquartile range, Non-IRA CTO—non-infarction-related artery chronic total occlusion, LVEF—left ventricular ejection fraction, DAPT—dual antiplatelet therapy.

**Table 3:** Cox proportional hazard regression analysis of the impact of clinical characteristics and non-IRA CTO status on cardiovascular death during follow-up.

Variable	Multivariate Cox Regression, Cardiovascular Death, HR (95% CI)
Age	1.020 (0.998–1.041)
Creatinine clearance	0.987 (0.977–0.997) *
Diabetes mellitus	1.631 (1.024–2.596) *
Peripheral artery disease	1.514 (0.908–2.523)
LVEF at discharge	0.950 (0.930–0.971) *
Syntax score	1.011 (0.984–1.039)
Low adherence to medical therapy	3.177 (1.755–5.752) *
Non-IRA CTO	0.886 (0.424–1.853)

Non-IRA CTO—non-infarct-related artery chronic total occlusion, HR—hazard ratio, CI—confidence interval, LVEF—left ventricular ejection fraction. * Statistically significant impact, *p* < 0.05.

**Table 4:** Clinical characteristics of 171 patients with non-IRA CTO in regard to CTO revascularization.

Clinical Characteristic, Median (IQR) or Number (%)		Non-IRA CTO Revascularized		*p* Value (Mann–Whitney or χ^2^ Test)
		Yes *n* = 51	No *n* = 120	
Age, years		66 (15)	71 (16)	0.078
Male sex		46 (90)	91 (76)	0.111
Creatinine clearance, ml/min		75 (35)	68 (38)	0.083
Arterial hypertension		42 (83)	98 (82)	0.906
Diabetes mellitus		23 (45)	37 (31)	0.088
LDL-C, mmol/L		3.4 (1.8)	3.3 (1.7)	0.668
Peripheral artery disease		10 (20)	30 (25)	0.672
Previous myocardial infarction		12 (23)	33 (27)	0.693
Previous PCI		10 (20)	27 (22)	0.866
Previous CABG		3 (6)	9 (8)	0.615
Previous stroke		2 (4)	11 (9)	0.304
Chronic obstructive pulmonary disease		4 (8)	6 (5)	0.573
Current smoking		23 (45)	60 (50)	0.718
Atrial fibrillation, any form		4 (8)	14 (12)	0.424
Body mass index, kg/m^2^		28.8 (6.9)	29.3 (5.2)	0.410
ACS type	STEMI	26 (51)	60 (50)	0.996
	NSTE-ACS	25 (49)	60 (50)	
Wrist vascular access		41 (80)	97 (81)	0.213
Left anterior descendent as IRA		15 (29)	56 (47)	0.082
Left anterior descendent as non-IRA CTO		25 (49)	32 (27)	0.024
Syntax score		28.5 (13.0)	26.5 (15.5)	0.447
Cardiogenic shock and/or cardiopulmonary resuscitation		4 (8)	11 (9)	0.592

Non-IRA CTO—non-infarction-related artery chronic total occlusion, PCI—percutaneous coronary intervention, IQR—interquartile range, CABG—coronary artery bypass graft, LDL-C—low-density lipoprotein cholesterol, STEMI—ST elevation myocardial infarction, ACS – acute coronary syndrome, NSTE-ACS—non-ST elevation acute coronary syndrome, IRA—infarct-related artery.

**Table 5:** Treatment strategies and long-term outcomes after discharge regarding CTO revascularization among 171 patients with non-IRA CTO found on initial coronary angiography for ACS.

Treatment and Outcome Variables, Number (%) or Median (IQR)		Non-IRA CTO Revascularized		*p* Value (χ^2^ or Mann–Whitney Test)
		Yes *n* = 51	No *n* = 120	
Complete revascularization		42 (82)	0	<0.001
LVEF at discharge, %		52 (13)	47 (20)	0.085
DAPT at discharge	Ticagrelor	28 (55)	82 (68)	0.467
	Prasugrel	6 (12)	12 (10)	
	Clopidogrel	17 (33)	25 (21)	
	No DAPT	0	1 (1)	
Statin at discharge	Maximal dose	49 (96)	114 (95)	0.853
	Submaximal dose	2 (4)	5 (4)	
	No statin	0	1 (1)	
Adherence to medical therapy after discharge	Low	11 (24)	31 (26)	0.181
	Moderate	15 (29)	57 (47)	
	High	24 (47)	32 (27)	
Cardiovascular death during follow-up		3 (6)	23 (19)	0.044

Non-IRA CTO—non-infarction-related artery chronic total occlusion, IQR—interquartile range, LVEF—left ventricular ejection fraction, DAPT—dual antiplatelet therapy.

**Table 6:** Cox proportional hazard regression analysis of the impact of non-IRA CTO revascularization and important clinical characteristics on cardiovascular death during follow-up among 171 patients with non-IRA CTO.

Variable	Multivariate Cox Regression, Cardiovascular Death, HR (95% CI)
Age	1.112 (1.045–1.184) *
Creatinine clearance	1.008 (0.986–1.030)
Diabetes mellitus	1.944 (0.795–4.755)
LVEF at discharge	0.914 (0.872–0.957) *
Non-IRA CTO revascularized	0.134 (0.017–1.041)
Non-IRA CTO of the left anterior descendent revascularized	0.218 (0.027–1.757)

Non-IRA CTO—non-infarct-related artery chronic total occlusion, HR—hazard ratio, CI—confidence interval, LVEF—left ventricular ejection fraction. * Statistically significant impact, *p* < 0.05.

## Data Availability

The data presented in this study are available on request from the corresponding author due to privacy and Ethics Committee restrictions.
